# Fundamentals of Edible Coatings and Combination with Biocontrol Agents: A Strategy to Improve Postharvest Fruit Preservation

**DOI:** 10.3390/foods13182980

**Published:** 2024-09-20

**Authors:** Marcela Miranda, Jinhe Bai, Lucimeire Pilon, Rosario Torres, Carla Casals, Cristina Solsona, Neus Teixidó

**Affiliations:** 1 Institute of Agrifood Research and Technology (IRTA), Postharvest, Edifici Fruitcentre, Parc Agrobiotech Lleida, Parc de Gardeny, 25003 Lleida, Spain; rosario.torres@irta.cat (R.T.); carla.casals@irta.cat (C.C.); cristina.solsona@irta.cat (C.S.); neus.teixido@irta.cat (N.T.); 2 US Horticultural Research Laboratory, United States Department of Agriculture (USDA)-ARS, Ft. Pierce, FL 34945, USA; jinhe.bai@usda.gov; 3 Embrapa Vegetables—Brazilian Agricultural Research Corporation, Brasilia 70351-970, DF, Brazil; lucimeire.pilon@embrapa.br

**Keywords:** postharvest disease management, shelf-life extension, water loss, gas barrier, fungal control

## Abstract

Challenges in global food supply chains include preserving postharvest quality and extending the shelf life of fruits and vegetables. The utilization of edible coatings (ECs) combined with biocontrol agents (BCAs) represents a promising strategy to enhance the postharvest quality and shelf life of these commodities. This review analyzes the most recent developments in EC technologies and their combination with BCAs, highlighting their synergistic effects on postharvest pathogen control and quality maintenance. Various types of ECs, including polysaccharides, proteins, and lipids, are discussed alongside coating fundamentals and the mechanisms through which BCAs contribute to pathogen suppression. The review also highlights the efficacy of these combined approaches in maintaining the physicochemical properties, sensory attributes, and nutritional value of fruits. Key challenges such as regulatory requirements, consumer acceptance, and the scalability of these technologies are addressed. Future research directions are proposed to optimize formulations, improve application techniques, and enhance the overall efficacy of these biocomposite coatings and multifunctional coatings. By synthesizing current knowledge and identifying gaps, this review provides a comprehensive understanding of the potential and limitations of using ECs and BCAs for sustainable postharvest management.

## 1. Introduction

By 2050, global agriculture will need to produce 50% more food, feed, and biofuel than it did in the last decade, owing to population growth [1]. This challenge requires increasing production using fewer resources while aiming to preserve nature and enhance farmers’ livelihoods. The agrifood system plays a crucial role in tacking global challenges across economic, environmental, and social domains [1,2,3,4].

Food loss and waste represent persistent global challenges across the entire supply chain, from harvest to retail and consumption. A global study conducted by the Food and Agriculture Organization of the United Nations (FAO) in 2011 revealed that 35–55% of produced fruits and vegetables are lost at the postharvest level [5]. Nearly a decade later, in 2019, FAO released the State of Food and Agriculture (SOFA) report, indicating that global losses continue to persist at approximately 33%. Enhanced postharvest management through innovative approaches, such as cutting-edge technologies and efficient logistics systems, can help address global challenges in mitigating these losses, particularly when aligned with sustainability objectives, including those outlined in the European Commission’s Farm to Fork strategy within the Green Deal initiatives, the United Nations Sustainable Development Goals (SDGs), and other international frameworks aimed at promoting sustainability [6].

An increasingly popular method for preserving fruit involves the application of a preserving food coating, notably using waxes such as carnauba and candelilla [7,8]. Waxes have been widely used as a fruit preservation technique, with their application dating back to the 12th and 13th centuries in China, particularly for preserving oranges and lemons through molten wax dipping, and later through brush application [9]. Waxing citrus fruits became common around the mid-20th century [10].

Edible coatings (ECs) have been employed in fruit preservation due to their film-forming properties, which establish a physical barrier around the product. This barrier can modify gas and water vapor exchanges, thereby altering the internal atmosphere of the fruit and preventing dehydration. The specific materials used in edible coatings—whether **proteins, lipids, carbohydrates, or composites** combining these elements—can influence their effectiveness in slowing the fruit ripening process and delaying physicochemical changes [11,12].

Accurate terminology is crucial when discussing filmogenic solutions derived from biopolymers capable of forming films. These biopolymers are large macromolecules consisting of repeating units, typically found in natural organic substances such as proteins, carbohydrates, and lipids [13].

These solutions should be called coatings, which are solutions applied directly onto the fruit surface and form a thin film on the fruit. In contrast, films are used to refer to them as a wrapping or packaging material [14,15]. The term biofilm should be used carefully to avoid misunderstanding, as it is used to refer to films cast from biobased polymer blends [16], whereas, in biological sciences, biofilm traditionally denotes a complex community of micro-organisms surrounded by a self-produced matrix or derived from the host (extracellular polymeric substances), which adhere to biotic or abiotic surfaces [17,18]. The term “edible coating” is only appropriate when the coating is safe to consume and is formulated with **Generally Recognized as Safe (GRAS)** substances. Using the proper terminology is crucial to prevent confusion and errors in its application and potential uses and to ensure food safety.

**Figure 1:**
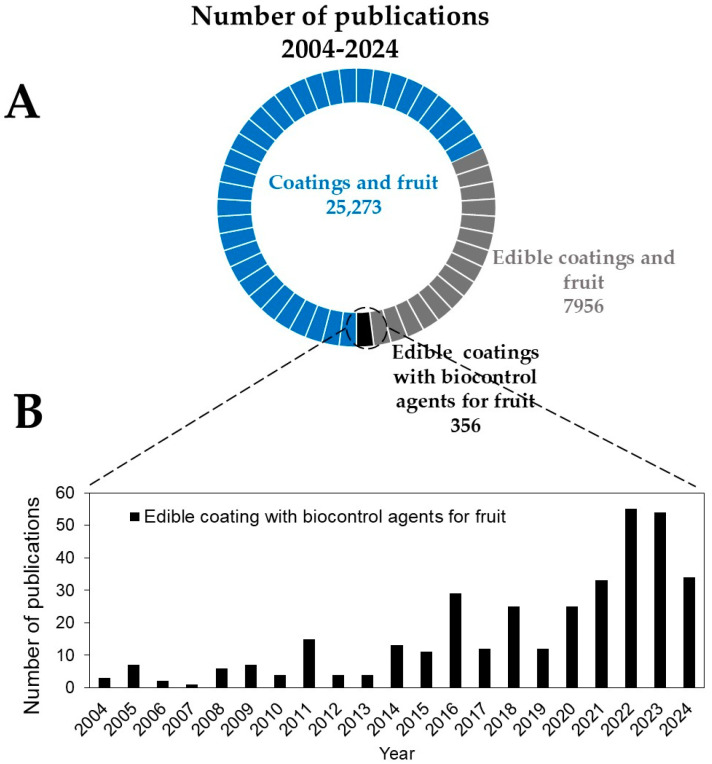
Distribution of publications concerning “coatings and fruit”, “edible coatings and fruit”, and “edible coatings with biocontrol agents for the fruit” from 2004 to 2024, sourced from the (**A**) ScienceDirect databases. Additionally, (**B**) displays publications related to just “edible coatings with biocontrol agents for fruit” over the past two decades. Data accessed in March 2024.

The volume of academic work in the coatings field in the last 20 years (2004–2024) is substantial, with approximately twenty-five thousand scientific and review articles published (Figure 1) according to ScienceDirect databases, indicating considerable interest in this technology. Figure 1 was created using this database for its extensive collection of peer-reviewed journals in the field and to provide an illustrative example of publication trends in terms of volume. However, other databases may also contain relevant studies that could further contribute to a more comprehensive understanding of the topic.

Edible coatings (EC) can be sourced from a variety of agricultural products, including corn, potatoes, rice, wheat, soybeans, and milk, among others. These coatings encompass a range of substances such as methylcellulose, hydroxypropyl methylcellulose, various starches, pectin, chitosan, collagen, gelatin, zein, gluten, casein, soy protein isolate, shellac, and beeswax, among many others [11,12]. Notably, EC formulations worldwide are increasingly focusing on utilizing agroindustrial waste, which totals over 2 billion tons and includes peels, seeds, sugarcane bagasse, coffee pulp, leaves, straw, oil cake, and other materials [3,4]. In response to these trends, nanotechnology has become crucial in advancing these coatings, significantly enhancing their properties and stability. These waste materials can be transformed into agrowaste-based nanoparticles using either top-down or bottom-up technologies and then incorporated into active coatings [19]. For example, extracting nanocellulose fibers (with an average diameter of 35 nm) from agricultural waste, specifically wheat straw, has demonstrated suitability for enhancing materials [20]. These fibers have potential for various applications, including but not limited to ECs and nanocomposites. A similar strategy was successfully utilized to develop nanocomposite coatings, resulting in an 8-day extension of bananas’ shelf life. This was achieved by applying a coating composed of chitosan, cellulose nanofibers extracted from garlic waste (skin), and nanocurcumin [21].

Building on these advancements, recent studies have explored specific applications of nanotechnology in fruit coatings. Studies have investigated the impact of chitosan nanoparticles on coated fresh-cut apples with a particle size of 110 nm. These studies have demonstrated a reduction in molds, yeasts, mesophilic, and psychrotrophic bacteria on the apple slices without compromising their quality attributes [22]. Nanomaterials, such as nanoscale carnauba wax emulsion with lipid micelles measuring approximately 44 nm, have exhibited beneficial properties for food items. These properties include facilitating optimal gas exchange, minimizing water loss, and enhancing the shine of ‘Nova’ mandarins and ‘Unique’ tangors [8]. Additionally, carnauba nanoemulsion-based coatings have been shown to reduce color development and slow the ripening of papaya fruit [23,24].

Nanotechnology enables the incorporation of various substances into a coating matrix, including antioxidant particles or natural extracts; probiotics and nutraceuticals; antimicrobial essential oils or nanoemulsion oils; and micro-organisms; as biocontrol agents (BCAs) for disease management. It also allows for the incorporation of isolated metabolites produced by these BCAs, as well as the use of micro and nanotechniques to preserve or control the release of payloads (such as essential oils, BCAs, interfering RNA, antioxidants, and nutraceuticals) [25,26,27,28,29,30].

In contemporary postharvest phytopathogen control, increasing attention is directed toward environmentally sustainable solutions, such as the application of essential oil vapors [31], with a notable interest in BCAs, such as bacteria and yeasts, the majority of yeasts, as alternatives to conventional chemical and synthetic treatments [32]. Extensively screened and formulated, these agents hold promise for controlling postharvest diseases; however, their practical implementation in commercial settings remains limited due to environmental stressors that reduce the viability of BCA cells and the enhancement of product handling, alongside additional challenges such as European regulation [33,34]. The narrow host and pathogen range that many biocontrol agents can target limits their commercial success. Genetic manipulation offers a potential solution to enhance biocontrol and broaden its application, though it presents significant regulatory challenges. Despite these obstacles, multinational companies are increasingly interested in biological control products, driven by rising concerns about pesticide resistance and the growing demand for “green” alternatives [32]. Significant progress has been made in the registration and commercialization of antagonistic micro-organisms for managing postharvest diseases caused by key phytopathogens such as *Penicillium expansum*, *P. digitatum*, *P. italicum*, *Fusarium sambucinum*, *Rhizopus stolonifer*, and *Botrytis cinerea*. Those commercial products have been introduced in countries including Belgium, the United States, Spain, South Africa, and the Netherlands [35].

The European Commission has undertaken efforts to decrease the overall use of chemical pesticides by 50% by 2030. This initiative highlights the significance of agricultural practices that reduce dependency on pesticides, enabling the introduction of pesticides containing biologically active substances. As part of the Farm to Fork (F2F) strategy, the Commission intends to revise the 2009 pesticide regulation and recognizes that new innovative techniques like biotechnology can enhance sustainability [6].

In this context, the combination of EC with BCAs offers a multifunctional solution, preserving postharvest quality while effectively managing postharvest pathogen decay [36]. This innovative approach aims to create a product solution that delays fruit ripening, enhances postharvest quality, reduces fungal decay, and extends shelf life. Novel multifunctional biopolymer coating could be an ecofriendly strategy to maintain fruit quality and reduce postharvest losses during storage [28,33,36,37].

Within this framework, studies have been conducted on the application of the biocontrol agent *Candida sake* CPA-1, formulated in a liquid solution combined with a commercial coating tested on grapes in the fields to control *Botrytis* spp. [38,39]. In later years, *C. sake* CPA-1 was formulated with two film-forming substances—potato starch and maltodextrin—to maintain cell viability when applied to grape surfaces, achieving phytopathogen control [34]. The BCA *Meyerozyma caribbica*, in combination with a sodium alginate coating, was used to coat avocados to mitigate *Colletotrichum gloeosporioides* (Pa14) infection and minimize weight loss. The efficacy of preventive treatments demonstrated better results than the curative method, consistent with established patterns [40]. Those studies evidence the prospective benefits of employing this strategy to enhance postharvest treatments.

Although studies on “edible coatings for fruits” have grown significantly over the past two decades (Figure 1A), research specifically on “edible coatings with BCAs for fruits” remains comparatively limited (Figure 1B). This disparity highlights the need for more studies focusing on multifunctional coatings that can control diseases while also delaying ripening. Advancing this technology is crucial for broader and more practical commercial applications. This review aims to explore the integration of ECs and BCAs as a sustainable approach to reducing postharvest losses in fruits and vegetables. We will examine the mechanisms by which ECs and BCAs work, their effectiveness in extending shelf life, and the challenges and future directions for their implementation in the agrifood industry.

## 2. Fundamentals and Applications of EC

The use of coatings has increased due to their capacity to enhance fruit storage. Notably, this technology does not seek to supplant established postharvest methodologies, such as the cold chain; rather, its objective is to complement them by prolonging shelf life and preserving fruit texture, nutrients, and freshness [18], while reducing physiological disorders like superficial scald and fungal diseases [41]. Furthermore, careful consideration of material composition and the fruit type to be coated is essential for achieving an optimal fit between the coating properties and the crop demands [18,42].

Coatings typically consist of thin layers of biopolymer (proteins, carbohydrates, and lipids) edible materials capable of forming a continuous film on the food/fruit surface [12]. The use of **GRAS materials** in fruit coating applications, particularly when the peel or skin is intended for consumption, is a regulatory imperative aimed at safeguarding consumer health and ensuring compliance with safety standards [18,43]. The components used should ensure consumer safety and adhere to high-quality international guidelines and regulations, including FDA approval (Food and Drug Administration) and the Codex Alimentarius Commission standards [44,45].

The degradability of the edible coating is a significant consideration in enhancing their environmental sustainability and functionality. Polysaccharide- and protein-based coatings are typically biodegradable, breaking down under composting or natural soil conditions, thus reducing waste and supporting a sustainable food supply chain [46]. Films made from polysaccharides—such as soluble starch, carboxymethylcellulose, sodium alginate, microcrystalline cellulose, pectin, carrageenan, potato starch, chitosan, and maltodextrin—exhibit notable biodegradability, with degradation rates of 55–80% after 10 days in natural soil [47]. This highlights their potential to enhance sustainability while retaining functional properties.

**Figure 2:**
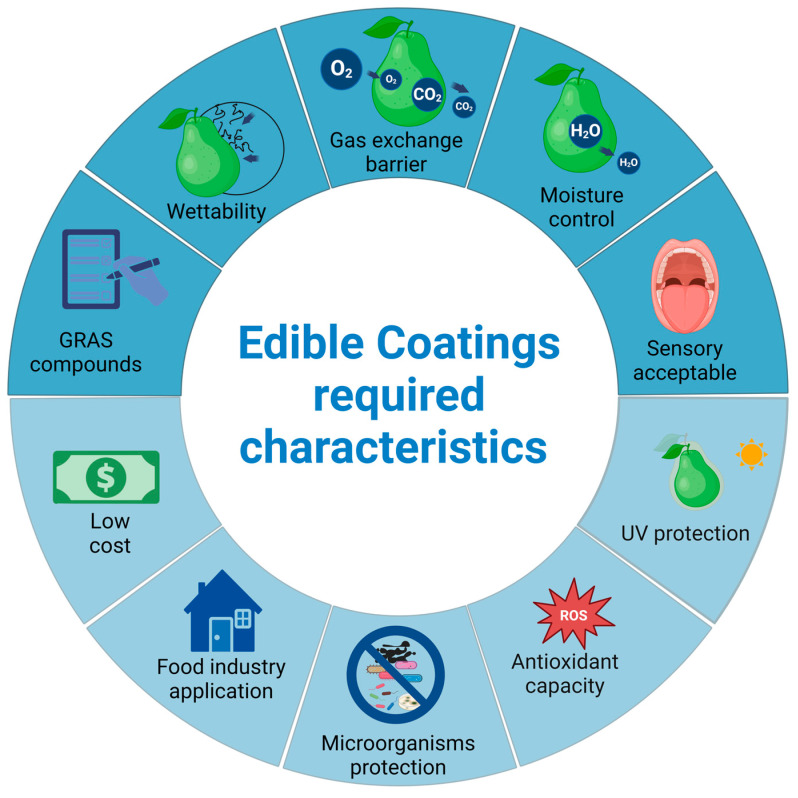
Essential (dark blue) and advanced (light blue) requirements of edible coatings for extending fruit shelf life and optimizing supply chain efficiency. Figure adapted from BioRender [51] templates.

To ensure the efficacy of coating material, it is imperative that it adheres to specific criteria delineated in Figure 2. These criteria include achieving low viscosity, using a film-forming solution with proper dispersion, and ensuring the coating is not sticky on the fruit surface. To achieve this, factors such as optimal **fruit wettability** to facilitate uniform surface coating and an ideal **gas exchange barrier** must be considered, taking into account the transmission of oxygen, carbon dioxide, **and water vapor**. Nevertheless, reduced oxygen levels could induce fermentative metabolism and thereby impart off-flavor to the fruit. Coatings **should not adversely impact the sensory attributes** of the product, and its taste ought to be deemed acceptable, using safe and healthy substances [8,11].

Regarding the gas exchange barrier, when formulating a coating, it is essential to ensure that the level of internal gases within the fruit is adequate [11]. Various mandarin hybrids coated with different materials, such as waxes (polyethylene and candelilla) and resin (shellac), were evaluated by sensory taste panels. It was observed that fruits coated with a low gas permeability coating (shellac) exhibited less fresh flavor compared to those coated with coatings having higher gas permeability (polyethylene and candelilla waxes) [48]. Authors demonstrated that the mandarin could be adversely affected by off-flavors under conditions where internal carbon dioxide exceeds 14%, internal oxygen falls below 4%, and juice ethanol content exceeds 1500 µL·L^−1^ after 7 days of storage at 20 °C [8]. In this scenario, preventing fermentative metabolism in coated fruit is crucial for maintaining sensory quality. It is an inefficient use of time and resources to extend the shelf life of fruit through treatments that ultimately lead to consumer rejection.

The application of coatings in specific scenarios has been shown to mitigate and prevent **physiological disorders,** such as superficial scald, which is brown or grayish-brown patches on fruit skin. Fernandez-Cancelo et al. [42] demonstrated this effect using a hydroxypropyl methylcellulose (HPMC)-based coating on ‘Granny Smith’ apples, which induced modifications, including ethanol accumulation and α-farnesene reduction. These changes contributed to reducing the superficial scald on coated apples by suppressing ethylene mechanisms that promote this disorder.

Whenever feasible, the incorporation of substances with **ultraviolet (UV) protection** (280–315 nm) [49], **antioxidant and antimicrobial properties** is advantageous; this includes organic acids, bacteriocins, lactoperoxidases, essential oils or their major compounds, and plant extracts, which have the potential to retard enzymatic modifications and reduce microbial growth [36,43,50]. Ideally, the implementation of coating **should minimally disturb packinghouses and industry systems**, considering their **low costs** compared to other alternatives [8,11,43].

### 2.1. Matrices and Functionality

**Figure 3:**
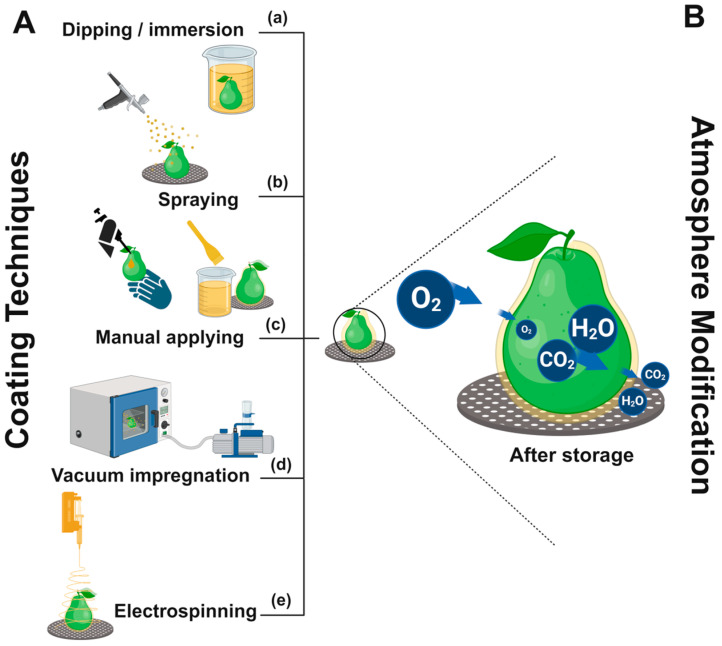
(**A**) Coating techniques for applying biopolymer dispersions on fruit surfaces: (**a**) Dipping: Fruits are immersed in the coating solution for an even surface layer. (**b**) Spraying: The coating is dispersed as fine droplets to form a thin layer. (**c**) Manual Application: A brush or similar tool applies the coating precisely to targeted areas. (**d**) Vacuum Impregnation: The fruit is placed in a vacuum chamber, where reduced pressure facilitates coating impregnation. (**e**) Electrospinning: An electric field induces the coating solution to form fine fibers, creating a uniform, nanometer-thin layer. All these techniques enable the formation of a coating that modifies the internal atmosphere of the fruit (**B**) by reducing gas exchange, thereby maintaining humidity and delaying ripening. Figure created on BioRender.com. Composite coatings, a growing category in the field, combine the advantageous properties of various materials to meet specific fruit preservation needs. This category includes nanosystem coatings that enhance properties like antimicrobial activity and controlled release, reduce residual taste, and increase solution stability through nanotechnology [23,27,46]. Producing structural biopolymers is a fundamental step in creating new materials that interact with food, fruit, and plant environments. It is crucial to highlight the significant advancements in developing advanced materials for healthcare and medical bioengineering, focusing on biocompatible materials suitable for human use, underscoring their importance for researchers in the coatings field aiming to enhance coating designs [25,53].

Traditionally, coatings are developed using hydrophilic and hydrophobic materials; hydrophilic coatings, characterized by the dominance of amino and hydroxyl groups, are charged either positively or negatively, attracting polar molecules like water. These materials demonstrate excellent solubility and uniform dispersion in water, which facilitates homogeneous film formation [18]. Such materials include polysaccharides like pectin, chitosan, alginate, starches, cellulose, and gums. Polysaccharide-based coatings generally do not provide effective barriers against water loss. However, their strong affinity for water helps maintain a hydrated appearance and enhances the surface’s shine [11,52]. Typically, hydrophobic coatings are composed of materials with electrically neutral structures and indistinct polar regions. In the presence of water, these materials tend to cluster and repel polar molecules. Common materials for hydrophobic coatings include proteins, oils, and fatty acids [18,52]. Protein coatings, such as zein, whey protein, sodium caseinate, and pea protein, and lipid coatings, including waxes like carnauba, candelilla, sugarcane, beeswax, and shellac, are examples. Typically, films from protein or lipid coatings serve as barriers against moisture, oxygen, and carbon dioxide, thus effectively delaying fruit senescence (Figure 3B) [11,28].

### 2.2. Application of Edible Coatings on Intact Fruits

Tailoring the application of ECs to specific needs and properties can lead to more effective and advantageous outcomes. Several techniques can be utilized, each with its own set of advantages and disadvantages. Generally, coatings are applied in a single layer to optimize processing time; however, layer-by-layer approaches are reported in the literature, although the commercial applicability of multilayer coatings remains uncertain [54]. 

The techniques include **dipping or immersion** (Figure 3a), **spraying** (Figure 3b), **manual application by hand or brushing** (Figure 3c), and **vacuum impregnation** (Figure 3d), among other approaches, such as **electrospinning application** (Figure 3e) [22,23,55,56,57]. Other methods, such as **fluidized-bed and panning**, are available. However, they are not commonly used on fruit surfaces [14]. Clean fruits to remove dirt and residues before applying the coating to minimize interference and ensure proper adhesion to the fruit surface is advisable [58].

**Figure 4:**
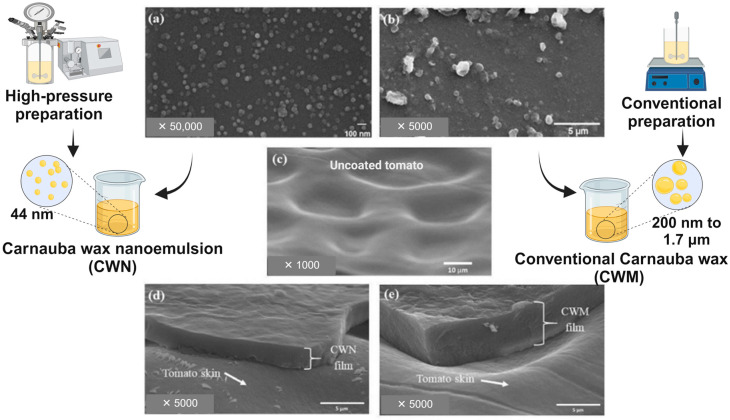
Interactions between coatings and fruit skin. Micrographs of the dispersion solution provide evidence of particle sizes (**a**,**b**), with variations in particle size achieved through different preparation techniques (conventional or high-pressure preparation). Images of uncoated tomato skin (**c**) and tomato skin coated with nano (**d**) or micro (**e**) coatings are presented, illustrating the differences in coating thickness. In both cases (**d**,**e**), the tomatoes were coated by dipping. This figure is reproduced with permission from Miranda et al. [44] and adapted using BioRender.com.

The **dipping or immersion technique** is commonly used on a laboratory scale. It involves submerging the fruit into a coating dispersion for a specific time to ensure proper coating deposition. The immersion period is critical for ensuring coating adhesion to the fruit surface. Time control and substrate interaction are key points to achieving an efficient and uniform layer. Other factors, such as speed of withdrawal, number of cycles, coating density, viscosity, surface tension, and drying conditions, should also be considered. A notable disadvantage of employing the dipping method for coating applications is the challenge of regulating the precise amount of coating deposited on the fruit surface, and controlling thickness is an important parameter that directly affects coating functionality. Particle size dispersion is also a factor that may influence coating thickness (Figure 4). Additionally, the necessity to drain excess solution can result in increased time consumption, potentially reducing its efficiency in commercial settings and causing cross-contamination [44,59]. Problems such as coating dilution, dirt buildup, micro-organism growth, and ticker layer may be caused by this application. Nevertheless, this method is employed when a complete surface coating is required on complex or uneven surfaces [58]. However, it represents a low-cost technique.

The **spraying technique** involves applying a thin coating layer onto the fruit surface via nozzles. The pressure utilized facilitates the atomization of the coating, a process wherein the liquid coating material is broken down into fine droplets. The distribution of droplet sizes in the sprays can be precisely controlled. This step is crucial to achieving an even and consistent distribution of the coating material on the target surface [58,59]. It is the most used method for applying coatings [58] on a commercial or semicommercial scale, especially in packinghouses where the application of wax emulsions on fruits like citrus is common. This method is well-suited for waxing citrus, followed by a hot air tunnel to allow the waxing to dry quickly [8,60]. Regarding the lab scale, this method is easy to simulate and makes experiments closer to the real application of these coatings using tools such as an airbrush [28], a garden pump spray bottle [22], or a backpack sprayer [39]. One advantage of the spraying technique is that the coating solution is less likely to become contaminated, as the solution is not exposed to multiple fruits and forms a more uniform thickness layer compared to the dipping method. However, a spinning system for the fruit is needed to ensure the entire fruit surface is coated. Additionally, a low-viscosity coating dispersion is required to prevent clogging of the nozzles, and the costs associated with control systems and operational nozzles may be high [58]. An alternative technique for applying a coating is **electrospraying**, which atomizes liquids through an electric potential difference, generating charged small droplets. This advanced spraying method is more efficient than conventional spraying and boasts superior transfer efficiency [59,61].

**Manual application methods**, including brush application, hand spreading, and latex hand coating, are frequently employed on a laboratory scale to optimize multiple formulations while minimizing material consumption. These techniques allow testing small volumes of formulated coatings, requiring only 0.5, 1, or 2 mL of the coating solution, depending on the fruit size. These methods facilitate rapid in vivo testing of formulations and enable the assessment of fruit appearance, a critical parameter for consumer acceptability. Moreover, it mitigates the risk of cross-contamination. A notable disadvantage includes the formation of a nonuniform thick layer on the fruit surface and the occurrence of foam due to abrasion, shear forces, and air introduction during the spreading process; however, a complete surface coating layer is typically achieved [8,59,62].

**Vacuum impregnation** is a relatively novel technique in the field of fruit coating. It is particularly innovative for application on intact fruits, as it removes water and air while simultaneously applying the coating dispersion. This system consists of a chamber where pressure is reduced using a vacuum pump and a recirculation system that continuously stirs the coating solution [55,63]. ‘Rich Lady’ peaches and ‘Prime Giant’ sweet cherries were treated with aloe vera gel using this method. A pressure of 0.2 mbar was applied for 5 min to coat the fruits. This resulted in reduced weight loss, minimized color changes, maintained firmness, decreased respiration rate, and reduced ethylene production after 40 days at 2 °C and 90% relative humidity (RH). These findings suggest that this method has the potential to be used in combination with coating design [63]. This method provides the advantage of rapidly infusing solvated compounds into plant tissues, particularly in fresh-cut fruit, to enrich the tissue with beneficial substances such as antioxidants, flavors, probiotics, and others. In contrast, studies of the vacuum pressure and treatment time are necessary to avoid causing mechanical damage to the fruit tissue [64].

The **electrospinning technique**, noted for its large surface area and porosity, high encapsulation efficiency, uniform morphology, low cost, and simple production, has garnered significant interest in the development of nanofiber films for food packaging [56]. A recent study has demonstrated its potential for direct application on fruit surfaces. Starfruits treated with a zein-based coating combined with jackfruit leaf extract using this technique were artificially inoculated with *Cladosporium tenuissimum* and *Aspergillus sydowii*. The coated fruits demonstrated a reduction in disease incidence and severity, indicating that this technique is a promising tool for encapsulating such extracts and enhancing postharvest management practices [57]. Electrospinning is considered a low-cost and efficient method for producing ultrathin nanofibers from a wide variety of materials [65]. However, despite these advantages, the final appearance of the fruit and its acceptance by consumers remain uncertain due to an opaque white layer, while the coating layer should be imperceptible or, in some cases, confer shine to the product.

It is important to emphasize that, in addition to the technical advantages and disadvantages discussed, the financial investment required and budget constraints to implement a technique must also be considered. For example, the citrus processing industry employs the spray technique [60]; it remains a widely used method since it is a well-established infrastructure and investments have been made since the 1950s. A range of specialized processes—such as degreening, washing, waxing, sizing, packing, and refrigeration—demand substantial capital investment and contribute to the overall cost of fruit preparation for the market. Nevertheless, these processes are critical for delivering fruit with uniform appearance, taste, and shelf life to consumers [10]. For the effective implementation of a technique, it is essential to carefully assess the balance between its benefits, associated costs—whether low, moderate, or high—desired company profit, and consumer demands.

### 2.3. Coating Deposition and Curing Process

**Figure 5:**
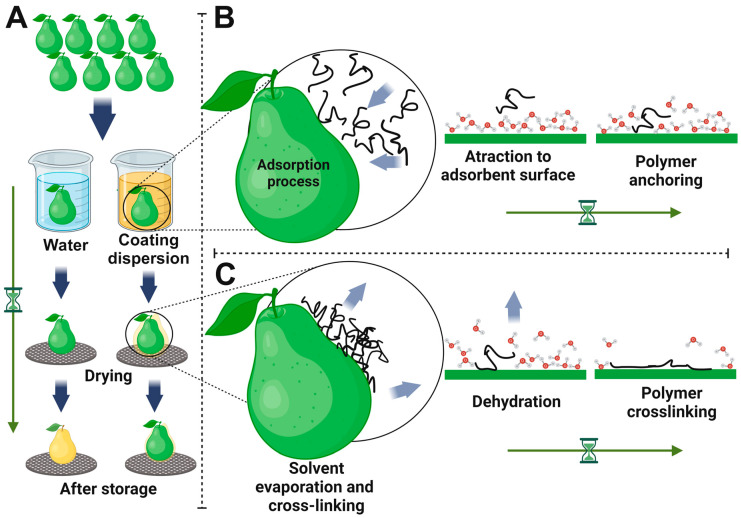
Schematic illustration of fruit coating deposition and curing process (**A**), detailing the adsorption process (**B**) and cure coating (**C**). (**B**,**C**) in this work were inspired and adapted by figures from Assis and Britto [18]. Figure created on BioRender.com.

The process of coating deposition on fruit surfaces involves extensive interactions until the coatings are effectively applied and adhered to the surface. Figure 5A depicts a schematic illustration delineating the steps involved in coating deposition on the fruit surface, exemplified by dipping application, resulting in the formation of a film after drying. When fruits are immersed in a coating dispersion, the biopolymers adhere to the fruit surface due to wettability and adhesion forces, establishing bonds of diverse strength—both weak and strong—with the fruit surface. The polymer, which is the adsorbate, is attracted to the adsorbent surface and anchored on the fruit surface (Figure 5B). The formation of films on fruit peel solutions involves the deposition of dissolved polymer species, which form bonds with the fruit surface. The proposed model emphasizes the influence of the adsorbent (fruit peel) and adsorbate (coating), and the interaction during immersion may include hydrogen bonding, hydrophobic interaction, dispersion forces, and electrostatic interaction to achieve the polymer anchoring [18].

The curing process involves the solidification of the coating to achieve its final properties (Figure 5C). Over time, the solvent gradually evaporates, initiating curing. This leads to the dehydration of the fruit surface, which allows the biopolymer to crosslink and form a protective film layer [18].

### 2.4. Coating Characterization

ECs and films are two distinct entities, which can occasionally lead to confusion. The first is applied in solution form directly onto the fruit surface, and once the solvent evaporates, it forms a thin film on the fruit. In contrast, the latter is used as a wrapping or packaging material [14,15]. ECs solutions can be characterized by viscosity, particle size, zeta potential stability, pH, color, and separation phase stability over time [44]. When the ECs solution is suitable for the lab casting method, including film formation and stripping, the formed film can be characterized by mechanical strength, elasticity, rheology, moisture and gas permeability, color, gloss, and light transmittance, all influenced by the biopolymer characteristics. Additionally, measurements can be conducted to determine thickness, moisture content, water solubility, contact angle or wettability, tensile properties, thermogravimetric analysis, Fourier transform infrared spectroscopy (FTIR) analysis, and X-ray diffraction [14,46].

All these parameters are crucial in achieving the optimal balance required to decrease internal oxygen levels and increase carbon dioxide concentrations within the fruit’s internal gas volume. This must be particularly managed to prevent the development of off-flavors, anaerobic respiration, or physiological disorders [14,44].

Understanding the relationship between the characteristics of the solution, the formed film, and coating functionality is essential, as the efficacy of ECs—particularly their oxygen and water vapor barrier properties—is highly dependent on the coating material matrix, as previously described. Carnauba wax (CW) exhibits a lower water vapor transmission rate compared to the chitosan coating material, thereby enhancing CW’s efficiency in preventing fruit weight loss. The functionality of a coating is influenced by the material properties and its affinity to the fruit surface [22,44]. Another example involves testing a bionanocomposite coating derived from an egg-based polymer on avocados, bananas, and papayas, revealing distinct contact angle measurements. These variations were attributed to the relatively higher hydrophobic waxy surface of papayas (contact angle ≈80°) compared to avocados (≈45°), while bananas exhibited intermediate wettability (≈60°) [66]. Consequently, it is imperative to acknowledge that the impact of the same coating may vary significantly across different fruits due to their distinct surface characteristics. Hence, detailed studies investigating the behavior of each fruit surface are essential, highlighting the improbability of developing a universal coating.

### 2.5. Mechanisms of Edible Coatings Action

**Figure 6:**
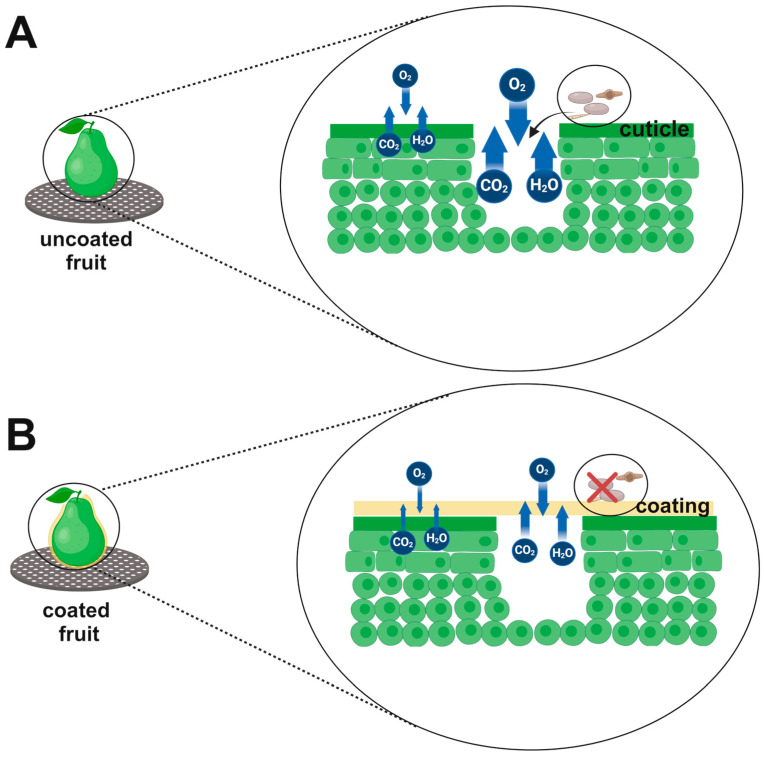
Schematic figure illustrating the effect of a coating on a porous region, such as stomata, lenticels, or wounds, establishes a physical barrier layer. (**A**) Uncoated fruits exhibit high levels of gas exchange, water vapor, and fungal infection possibility. (**B**) The edible coating formed on the fruit cuticle shields the fruit from infection. It reduces the interchange of gases and water vapor between the internal and external environments, thus slowing down metabolism. Figure 6 in this work was inspired from Assis and Britto [67]. Figure created on BioRender.com.

Coatings can create a physical barrier layer, clogging or protecting stomata, lenticels, and injuries [67]. They can also cover porous areas such as the stem scar, leading to fewer pathways for gas exchange [44,68] and pathogen infections (Figure 6). Edible materials may confer barrier properties against both moisture and gas interchange throughout the storage period, thereby retarding fruit respiration and senescence. However, cracks and detachments can reduce effectiveness by increasing gas exchanges [11]. This functionality serves to protect tissues from browning, discoloration, and texture softening [69]. In the case of fruits, low concentrations of oxygen and high concentrations of carbon dioxide, which do not cause stress to fruit metabolism, have the beneficial action of reducing respiration rates and ethylene production. Consequently, changes associated with ripening and senescence, such as the development of color, loss of firmness, and nutritional quality, are slowed down [70].

In this context, a coating must provide an effective gas barrier, balancing oxygen and carbon dioxide permeability to regulate fruit respiration and prevent anaerobic processes. Due to the varied respiration rates of different fruits, which require specific minimum oxygen transfer rates to avoid undesirable metabolic changes [69,71] it is crucial to consider the following aspects: (a) Gas exchange ratios—when the coating establishes appropriate gas exchange ratios, ethylene production is reduced in low-oxygen atmospheres (below 5%) due to the inhibition of ACC oxidase activity; (b) In high-carbon dioxide environments: in conditions above 1%, ACC synthase and ACC oxidase activities are inhibited; these enzymatic systems are vital for ethylene production, which influences ripening, senescence, and stress responses and (c) Off-flavor compounds—in atmospheres with less than 0.5% oxygen and more than 20% carbon dioxide, the fruit tends to develop off-flavor compounds such as acetaldehyde, ethanol, and ethyl acetate [70].

ECs such as carnauba in nano and microemulsion forms, alongside shellac protective coatings, were applied to citrus fruits (‘Nova’ mandarins—*Citrus reticulata* and ‘Unique’ tangors—*C. reticulata × C. sinensis*) and stored in cold (14 days at 10 °C) storage, followed by exposure to marketing conditions (7 days at 20 °C). They demonstrated reduced water loss and increased CO_2_ levels with lower O_2_ production. Carnauba coatings exhibited less ethanol and volatile profile alterations compared to shellac coatings. The authors suggest that carnauba coatings were as effective as, or potentially superior to, carnauba microemulsions in enhancing shine and flavor [8]. A polyvinyl alcohol-native potato starch coating incorporating carvacrol was used to treat ‘Golden Delicious’ apples and store them for 14 days at 25 °C and 65% RH. The coating application did not preserve fruit quality, such as weight loss, firmness changes, or respiration; however, it showed a significant black and green mold control due to the carvacrol antimicrobial activity [45].

Coatings could also activate mechanisms of host defenses. For example, chitosan coatings, a biopolymer coating material with antimicrobial properties, have noteworthy mechanisms of action, including (1) chitin fragments, a common sign that indicates a fungi or insect attack, which is recognized by the plant and starts the defense reactions. Chitosan and its fragments undergo a similar mechanism [72]. (2) The main action is based on the electrostatic interaction between positively charged amine groups that bind to the negatively charged surface of the micro-organism cell wall, causing osmotic damage and resulting in cell death. However, over time, as the microbial population increases, the number of available charged sites on chitosan remains constant. Consequently, after several days of fruit storage, all chitosan binding sites become saturated, thereby diminishing its overall antimicrobial efficacy [22]. (3) Similar mechanisms of cell disruption are described based on the chitosan chelating capacity to interact with metal ions in the bacterial cell surface, resulting in the rupture of micro-organisms. (4) Chitosan can disrupt cellular functions by altering transcription and translation processes [73].

Table 1 demonstrates some materials employed in recent research endeavors concerning fruit coating, aiming to preserve fruit freshness and extend shelf life. It is worth noting that the research on ECs extends beyond their use for shelf life storage (20–25 °C and 80–95% RH for 4–7 days) and includes testing under cold storage conditions. Among all the coatings, there has been a significant increase in publications regarding chitosan-based coatings. As of June 2024, a search on ScienceDirect using the terms “chitosan”, “coating”, and “fruit” yielded 8874 scientific articles, with 2844 of these articles published in 2023 and 2024. Table 1 presents additional examples of effective coating applications in post-harvest fruit quality management, highlighting their substantial potential to improve shelf life and preserve fruit quality. These examples are collected from various sources, including ScienceDirect, though they are not limited to this platform.

## 3. Biocontrol Agents for Postharvest Disease Management

### 3.1. Enhancing Postharvest Disease Management by Biocontrol

The term “biological control” or “biocontrol” should be applied when referring to the utilization of living agents, including viruses (though they may not always fit the definition of living organisms), to mediate and manage harmful organisms such as pathogens, pests, and weeds, to achieve various benefits for humans and the environment. It is essential to maintain conceptual and terminological clarity in this context. Nonliving and nature-based substances offer distinct forms of bioprotection and should not be referred to as biocontrol. It is crucial to uphold a distinct conceptual delineation between these entities for scientific and regulatory purposes [80].

In recent years, progress in understanding crop diseases and biocontrol strategies has increasingly emphasized studying the fruit microbiome. This approach challenges the traditional single pathogen-disease model by highlighting that disease development can involve complex interactions between a pathogen and the micro-organisms already present at the wound site. Current research in this area is focused on how the microbiome can influence the disease process, aiming to achieve a more comprehensive and holistic perspective on crop disease management [81]. Various aspects, including the interaction system of the antagonist, the microbial communities on the fruit surface, host responses, and pathogens, contribute to advancing a deeper understanding of how to formulate and apply this micro-organism effectively [35].

The traditional paradigm of disease caused by a single pathogenic organism to initiate an infection has been reconsidered, leading to the exploration of the concept of pathobiome. Pathobiome represents a combination of microbial species associated with an organism interacting with each other and the host to facilitate disease development. Consequently, the entire microbial community is implicated in either promoting or inhibiting the progression of infection and disease [81,82,83]. 

In this text, we focus on harnessing the power of micro-organisms to reduce fruit postharvest diseases primarily caused by fungi. BCAs are commonly used to control fruit pathogens, and some examples include bacteria (e.g., *Pantoea* spp. [84,85], *Pseudomonas* spp. [86], *Bacillus* spp. [86,87]); yeasts (e.g., *Candida* spp., *Pichia* spp. [88,89]); fungi (e.g., *Trichoderma* spp. [90]); yeast-like fungi (e.g., *Aureobasidium pullulans* [91]); and viruses (e.g., bacteriophage cocktail, including phages such as *Salmonella* Enteritidis F5–4, *S.* Typhimurium L2–1, and *S.* Typhimurium ICB1–1 [92]. The framework focuses on postharvest applications for fruit. However, it is essential to consider that preharvest treatments also influence postharvest management and should be integrated into the planning process. Since infections predominantly originate in the field, effective postharvest disease control strategies must be initiated at the field level [93].

The utilization of biocontrol in preharvest practices has significantly increased worldwide. A search conducted in June 2024 for the terms “pre-harvest and biocontrol” on ScienceDirect over the past five years yielded 1004 research articles and 272 review articles, demonstrating a substantial interest in this mechanism. In preharvest, studies with BCAs formulated without coating forming matrices are dominant; however, studies have explored the use of BCAs combined with coating formulations to enhance field disease control and improve fruit quality, thereby preventing both quantitative and qualitative losses [38,39]. Grapes cultivated for organic wine production were treated with *C. sake* CPA-1 in combination with a fatty acid-based coating, marketed as Fungicover^®^ to control botrytis bunch rot. This treatment, applied between flowering and harvest, significantly reduced *Botrytis* bunch rot incidence and severity [38]. Additionally, the treatment demonstrated robust field survival and persistence of *C. sake* [39]. These findings support the implementation of alternatives to synthetic fungicides for effective and practical disease management. 

Postharvest biocontrol systems entail a complex tripartite interaction involving antagonist microorganisms, the pathogen, and the host, which is intricately influenced by environmental conditions. This dynamic interplay underscores the significance of understanding these factors to enhance the efficacy and reliability of biocontrol strategies in preserving postharvest quality and safety of agricultural produce [94]. In the context of postharvest studies focusing on “postharvest and biocontrol”, a search on ScienceDirect over the past five years resulted in 589 research articles and 173 review articles using these terms.

Studies have showcased the effectiveness of antagonist bacteria and yeasts in mitigating postharvest diseases in tomato fruit. One notable example involves the application of six antagonistic bacteria (*B. subtilis*, *B. amyloliquefaciens*, *P. resinovorans*, *P. alcaligenes*, *P. putida*, and *P. stutzeri*) to control *Geotrichum candidum* and *Alternaria alternata* on tomato fruit during two harvest seasons. The results indicated a reduction in *G. candidum* incidence ranging from approximately 90% for fruits treated with *B. subtilis*, *B. amyloliquefaciens*, or *P. stutzeri* across two seasons (2021 and 2022). For *A. alternata*, the results demonstrated an approximate 80% reduction in incidence for fruits treated with *B. subtilis* and *B. amyloliquefaciens* [86]. Another study regarding postharvest disease management on tomato fruit using *A. pullulans* S2, a yeast-like fungus, effectively reduced the incidence of natural decay on ’Provence’ tomatoes after 12 days of storage [91].

Another study evaluated the efficacy of *Trichoderma*, *Bacillus* strains, and their extracts in protecting mangoes against *Lasiodiplodia theobromae*, and *C. gloeosporioides*. The results demonstrated that *Trichoderma*, *Bacillus*, and the extracts reduced disease incidence and severity. Regarding the fruit quality, the treatments did not affect any parameters [90]. *Metschnikowia pulcherrima* was effective against *B. cinerea*, *P. expansum*, *Alternaria* sp., and *Monilia* sp. on ‘Golden Delicious’ apples, achieving control levels against *B. cinerea* and *P. expansum* comparable to those observed with the chemical treatment thiabendazole [95].

The application of *C. oleophila* to either wounded or intact grapefruit surfaces has been shown to induce resistance against one of the most problematic citrus pathogens, *P. digitatum*. This treatment resulted in effective protection against citrus decay [96]. A yeast species identified as *Candida pruni* sp. nov. has shown potential as a biocontrol agent against brown rot caused by *Monilinia fructicola* on peaches. Collected from plums, this yeast demonstrated the ability to reduce disease incidence from 100% to 27% compared to untreated, inoculated control fruit [97].

The antagonistic microorganism *M. guilliermondii* LMA-Cp01, which possesses a broad spectrum of activity, was tested against several postharvest pathogens, including *A. alternata*, *P. italicum*, *P. digitatum*, *P. expansum*, *B. cinerea*, and *R. stolonifera*. The results demonstrated an inhibition of incidence and severity ranging from 30% to 85% on fruits such as persimmons, oranges, apples, and pears [98].

A yeast strain of *P. anomala* (strain K) has demonstrated antagonistic effects against *B. cinerea* in apples and has been characterized by its mechanisms of fungal suppression. One mechanism involves the production of glucanases, specifically Exo-β-1,3-glucanase, which induces morphological changes in the fungal cells, such as cytoplasmic leakage and cell swelling [89]. The susceptibility of bananas to the complex crown rot disease is caused by the association of three fungi—*C. musae* (Berk. & Curt.) Arx, *F. moniliforme* Sheldon, and *Cephalosporium* sp.—were found to be strongly inversely correlated (r^2^ = 0.83) with the protection conferred by two yeast strains, *P. anomala* strain K and *C. oleophila* strain O. These findings suggest that these yeast strains have significant potential in protecting bananas from crown rot disease [88].

Research on the microbiome, microbial consortia, and the implications of recent paradigm shifts is relatively recent. These studies hold the potential to support information the development of novel strategies for manipulating microbial communities [81,82]. Given the promising outcomes demonstrated, continued research in biological control is imperative for advancing sustainable agricultural practices and effectively managing crop diseases.

### 3.2. Mode of Action of Biocontrol Agents

The mode of action of antagonistic microorganisms can be attributed to several mechanisms, including competition for nutrients and micronutrients such as iron, space, biofilm development, mycoparasitism, production of secondary metabolites, volatile organic compounds, lytic enzymes, peptides, antibiotics, and induction of defenses. Depending on the microorganisms, one mechanism may be more prevalent than another [82,94,99,100].

Antagonist yeasts use volatile compounds and produce cell-wall degrading enzymes like chitinase, cellulase, protease, and glucanase, among other mechanisms [43,101]. Numerous bacterial genera, including *Bacillus*, *Pseudomonas*, *Streptomyces*, *Pantoea*, *Lysobacter*, and *Enterobacter*, play a key role in producing metabolites that can control other microorganisms. *Bacillus* and *Pseudomonas* are widely recognized for their powerful antifungal properties against postharvest fungal pathogens, as they produce metabolites that are effective antifungal antibiotics in combating pathogens [94,99,102].

Other genera, such as *Starmerella*, including *S. bombicola* species, produce biosurfactants known as sophorolipids, which are stable, biodegradable, and low in toxicity. This biosurfactant can deteriorate the pathogen’s cell membrane, causing cell disruption and death. A study has shown the antimicrobial potential of these biosurfactants to control *B. cinerea* on tomato fruits [100]. However, it is important to emphasize that BCAs primary mode of action remains competition for nutrients and space. In this approach, the microorganism quickly consumes nutrients in the wound and produces substances like biofilms, which block the development of pathogenic spores [82]. Antagonistic microorganisms can act through parasitism, mycoparasitism, or hyperparasitism by attaching to the hyphae of fungal pathogens and producing extracellular cell wall lytic enzymes. These enzymes disintegrate the pathogenic fungal cell wall and inhibit spore germination [94,103]. Researchers examined the efficacy of 87 isolates of *P. guilliermondii* yeast obtained from apples against *B. cinerea* and *P. expansum*, finding that this yeast attached and collapsed the fungal hyphae. They demonstrated that the adherence of *P. guilliermondii* to *B. cinerea* hyphae could be delayed by agents that modify protein integrity such as salts, proteases, and specific sugars. The yeast produced 1,3-glucanase when cultivated using various carbon sources and on the cell walls of fruit pathogens. Cell-free supernatants from *P. guilliermondii* exhibited two to five times greater 1,3-glucanase activity compared to non-effective yeast used as control, suggesting robust attachment, combined with the secretion of cell wall-degrading enzymes, likely contributes to the biocontrol efficacy of this yeast antagonist [103].

In summary, BCAs provide an alternative method that utilizes multiple modes of action. They significantly reduce the risk of resistant strain development and act synergistically by establishing an interaction with the host, thus reducing the emergence of resistance [99]. Consequently, while this system holds significant promise for widespread application in the agricultural industry as an alternative to synthetic chemical pesticides, it is essential to evaluate each case and application individually.

## 4. Combining Edible Coating and Biocontrol Agents

ECs represent a cost-effective and environmentally low-impact method for extending the shelf life of fruits. Enhancements can be achieved by incorporating antagonistic microorganisms, reinforcement particles such as nanostructures, and various natural substances. Furthermore, when combined with appropriate storage conditions, this sustainable approach may significantly improve fruit quality [8,104]. An optimal coating should be multifunctional, addressing not only the control of fruit diseases but also regulating fruit ripening and providing protection against physiological disorders. Achieving this balance, however, remains a significant challenge.

Notably, research on integrating BCAs and ECs primarily concentrates on enhancing BCAs viability and managing postharvest diseases. Furthermore, a limited number of studies have showcased enhancements in these coatings during postharvest storage, such as delayed respiration of fruits and alterations in metabolic physiology. Hence, a deeper comprehension of this subject is essential to achieve expertise and develop feasible products for integration into the postharvest chain.

### 4.1. Relationship between Coating Matrix and Biocontrol Agents

The integration of the coating matrix with the microorganism can confer physical protective benefits and improve the survival rates of BCAs. Moreover, it offers additional benefits by reducing the physiological degradation of fruit [104,105].

Advantages associated with the coating matrix and BCAs include physical protection, as the coating matrix may provide a protective barrier around the microorganism [105]. Additionally, adherence and persistence are enhanced by matrices, improving the adherence of BCAs to surfaces, which increases the prospect of effective colonization or infection [40,105,106]. Properly designed coating matrices can extend the shelf life of BCAs, preserving their viability and effectiveness until application [106].

**Figure 7:**
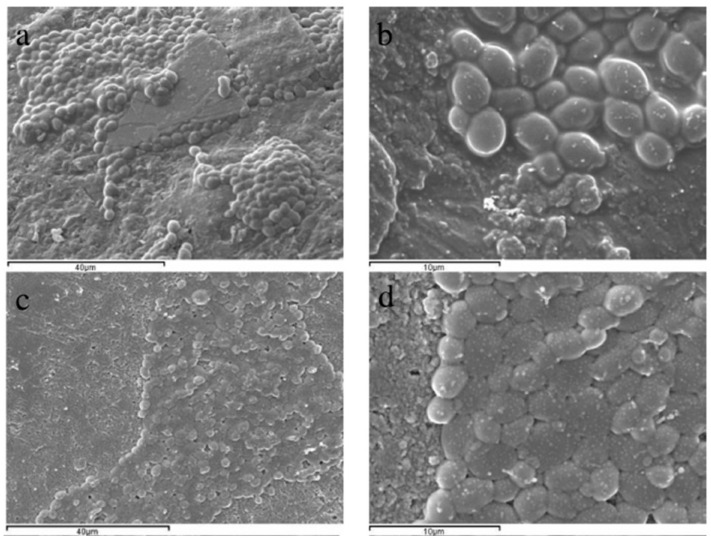
Micrographs of the surface appearance of grapes coated with *C. sake* dispersion on water (**a**,**b**) or formulated with sodium caseinate (**c**,**d**). Figure reproduced with permission from Marín et al. [28].

Coatings may enhance the efficacy of antagonist microorganisms through supplementation with additives, such as nutrients, that enhance the efficacy of BCAs. A study has shown positive outcomes, indicating that the coating matrix can serve as BCA nutrients and support the uniform spread of microorganisms (Figure 7), resulting in successful fruit surface colonization [28]. The interaction between the microorganism cell wall and the coating substance is critical to ensuring compatibility. Coatings permit homogeneous distribution of the BCAs on the pericarp and entrap the BCA [40,105]. Incorporating probiotic microorganisms, such as *Lactobacillus rhamnosus* or *Bifidobacterium animalis*, into alginate-based coatings enriched with oligofructose and inulin prebiotics has shown promising results. When applied to fresh-cut apples, these coatings reduced *Escherichia coli* growth and exhibited bactericidal activity against *Listeria innocua*. The treatment also effectively decreased yeast and mold levels after 8 days of storage at 5 °C while maintaining the nutritional quality of the apples [107].

Research on the use of bacteriophages on sliced cucumbers, apples, and whole cherry tomatoes found that applying an edible whey protein isolate coating improved the stability of the viruses during cold storage. In this study, *E. coli* growth was reduced by 2 logs (CFU) on apples and tomatoes. The coating not only facilitated phage loading and stability but also preserved antimicrobial activity, highlighting its potential for extending phage therapy to fresh produce [106].

Incorporating *M. caribbica* into a sodium alginate-based coating has enhanced avocado quality by delaying firmness loss and preventing internal flesh damage and browning during cold storage, followed by market conditions. Thermal analysis of the films revealed that the coating matrix protects *M. caribbica* while increasing the puncture force of the films containing the biocontrol agent. The addition of the yeast also reduced the coating’s thickness, water vapor permeability, and moisture content [105]. Further research demonstrated that applying *M. caribbica* within a sodium alginate coating significantly decreased the incidence of *C. gloeosporioides* in avocados, with greater efficacy observed when used preventatively rather than curatively. This effectiveness is attributed to the production of volatile organic compounds by *M. caribbica* and their likely entrapment within the coating matrix [40].

Regarding regulatory markets, the use of ECs requires the use of GRAS substances [44]. However, any new ECs containing BCAs must meet several safety requirements concerning biological agents, above all the Qualified Presumption of Safety (QPS) status from the European Food Safety Authority (EFSA) within Europe [108,109]. The “list of microorganisms with QPS status”, established in 2007, has been revised and updated annually until 2014, and since then, updates have been conducted and published triennially. When new information is reclaimed from literature searches that would modify the QPS status of a taxonomic unit or its qualifications, this information is published in the Panel Statement covering the preceding 6-month period [108]. BCAs can be consulted in the updated Excel file list of “QPS status recommended biological agents” adopted in December 2023 and available on the EFSA webpage [110]. 

The coating matrix is a crucial element in the formulation of biocontrol products. It ensures the protection, stability, and effective adherence of BCAs. While it may not always directly enhance overall performance in disease management, it significantly contributes to maintaining fruit quality. A comprehensive understanding and optimization of the relationship between the coating matrix and BCAs are essential for developing effective and sustainable multifunctional ECs. 

### 4.2. Synergistic Effects and Impact on Fruit Quality and Postharvest Pathogen Control 

Studies combining ECs and microorganisms may not be groundbreaking, but conducting them to develop a product that effectively controls fungal diseases and maintains fruit quality is essential. A study tested different coatings of shellac-based, sucrose esters, and surfactants combined with *C. oleophila,* which were tested on grapefruit stored at 13 °C for 90 days. The authors enhanced the survival of biocontrol yeast and increased colonization on the fruit surface. The formulation use of sucrose ester combined with *C. oleophila* showed promising results in extending fruit shelf life by reducing decay incidence [111]. Edible coatings formulated from *Lallemantia royleana* mucilage enriched with *Lacticaseibacillus casei* significantly improved the sensory attributes and extended the shelf life of fresh pistachios by mitigating fungal growth, weight loss, and lipid oxidation. Moreover, they increased chlorophyll and phenolic content, along with enhancing antioxidant activity during a 35-day storage period, in contrast to uncoated fruit. The probiotic-coated pistachios were highly accepted by panelists [112].

Research has shown that incorporating lactic acid bacteria (*L. plantarum*) into different ECs matrices, such as pregelatinized potato starch-based or sodium caseinate-based, effectively controls fungal growth and maintains the postharvest quality of grapes. The sodium caseinate-based coating improved the survival of *L. plantarum* on grape skin after seven days of storage compared to fruits coated with microorganisms dispersed in water. Additionally, the potato starch-based coating combined with *L. plantarum* reduced *B. cinerea* incidence more effectively than the sodium caseinate-based coating loaded with BCA or dispersed in water [37].

The utilization of coating matrices with inherent antimicrobial properties has been increasingly employed alongside biocontrol agents (BCAs) due to their diverse range of characteristics. A chitosan-based EC combined with a bacteriophage applied to tomatoes reduced *E. coli* growth after 6 days of storage at 20 °C. The phage showed stability and activity over a period of one week and decreased bacterial growth by 3 logarithmic units [113].

The microorganism *C. sake* was formulated using fluidized-bed spray-drying with a combination of maltodextrin, skimmed milk, sucrose, or pregelatinized potato starch to create a film-forming product that evenly distributes the BCA cells and enhances survival. The formulations were characterized and evaluated for their efficacy in controlling *B. cinerea* on inoculated grapes stored at 20 °C and 85% RH for 10 days. The pregelatinized potato starch coating combined with *C. sake* reduced fungal severity by over 65%, while the maltodextrin coating and *C. sake* formulation reduced approximately 40% [114].

Commercial implementations of ECs incorporating BCAs remain limited in scope. However, research on extracting and isolating antimicrobial compounds from BCAs and integrating them into EC matrices has steadily grown. BCA metabolites like peptides, toxins, and antibiotics are being used to enhance the coatings’ effectiveness [78,115].

BCAs metabolites such as sophorolipids from *Starmerella bombicola* and fructooligosaccharides from *B. subtilis* were incorporated into a cassava starch biopolymer coating to treat fresh strawberries. The treated samples were analyzed over a period of 15 days at 4 °C and 85% RH. Sophorolipids display antimicrobial properties. Furthermore, fructooligosaccharides can enhance the prebiotic characteristics of the coating. Including both metabolites in the coating demonstrated superior performance by extending fruit shelf life, controlling mold and yeast growth, and preserving the nutritional content of the strawberries [78].

Another study used peptide-based ECs to manage postharvest fungal infections. The researchers utilized bioactive peptides derived from the fermentation of palm kernel cake—a by-product of oil extraction—employing *L. plantarum* and *L. fermentum*. They prepared film-forming solutions with 10 different polysaccharides as coating matrices such as chitosan (2.5%), potato starch (2%), pectin (3%), carrageenan powder (1%), microcrystalline cellulose (2.5%), inulin (3%), sodium alginate (1.5%), soluble starch (2%), maltodextrin (3%) and carboxymethyl- cellulose (1%) (*w*/*v*), combined with 20 mg mL-1 of peptide from each Lactobacillus. The addition of peptides to chitosan resulted in significant improvements in inhibiting the severity of *C. gloeosporioides* and *Botryodiplodia theobromae*, as observed through in vitro and in vivo assays using ‘Susu’ mango fruits, compared to chitosan alone or untreated fruits, after 3 and 6 days at 25 °C. Additionally, the presence of peptides caused noteworthy changes in the color and water vapor permeability properties of the polysaccharide-based films [47].

Further research has shown that using a chitosan-based coating with added peptides can help preserve mangoes for longer storage. Mangoes coated with peptides-chitosan-based coatings have an extended shelf life of 17–18 days, compared to 14 days for mangoes coated only with chitosan and 9 days for uncoated mangoes. Quality factors such as firmness and weight loss also differ significantly between coated and uncoated mangoes [115].

A cassava starch-based coating enriched with sophorolipid and fructooligosaccharide from microorganisms was used to maintain strawberry quality, which was monitored over 15 days at 4 °C and 85% RH. This treatment effectively delayed fruit senescence, reduced weight loss and anthocyanin content, and inhibited the growth of mold and yeast, suggesting that such coatings could extend the shelf life of highly perishable fruits [78].

Table 2 presents a comprehensive summary of recent research on BCAs integrated into coating dispersions for the preservation of fruits. Each entry details the antagonist used, the specific fruit, the matrix or coating materials and additives employed, the targeted pathogen, storage conditions, and key findings. The studies cover a range of biocontrol strategies, from bacteriophages and yeast species to lactic acid bacteria and other microbial agents, highlighting their efficacy in mitigating various pathogens and extending the shelf life of fruits.

## 5. Trends and Challenges in Edible Coatings Incorporating Biocontrol Agents

Several factors influence the development and effectiveness of multifunctional coatings that integrate ECs with BCAs, including the coating composition, BCA concentration and stability, and the interaction between these components. Additionally, environmental conditions like temperature, humidity, and microbial load present significant challenges to maintaining the efficacy of these treatments.

Scaling up the use of these technologies for widespread adoption poses significant challenges related to maintaining production consistency and cost-effectiveness, as well as ensuring uniform application across various products. Addressing these challenges necessitates additional rigorous investigation into practical and commercial applications. Advancements in nano and composite ECs enriched with BCAs, as well as encapsulation or nanoencapsulation with additives, show promise in enhancing the stability and activity of these treatments.

Regulatory frameworks and guidelines exhibit significant global variability, emphasizing the need to ensure the safety of the components. A thorough understanding of diverse approval processes underscores the urgent requirement for international standardization and harmonization. This unified approach will facilitate widespread adoption and enhance global acceptance of these innovative solutions.

## 6. Conclusions

Addressing the increasing demands on global food production while reducing postharvest losses is essential for achieving food security and sustainability. Multifunctional products are based on ECs incorporating BCAs present natural and efficient alternatives to chemical treatments. These innovations have the potential to reduce dependency on hazardous pesticides and preservatives while simultaneously enhancing the shelf life and safety of perishable commodities. ECs create a protective barrier, slowing respiration, reducing moisture loss, and inhibiting microbial growth. When combined with BCAs targeting postharvest pathogens, typically fungi, their effectiveness can be significantly enhanced, while improving the quality and shelf life of fruits and vegetables. Integrating agro-industrial waste materials into coatings aligns with efficient resource management principles, adding value to waste and promoting sustainability. Nanotechnology and genetic engineering offer significant potential for advancing the effectiveness of EC. However, challenges remain, including optimizing coating formulations, obtaining regulatory approval, and achieving consumer acceptance. Further research is needed to optimize these coatings tailored for different types of produce, ensure their safety, and evaluate their economic viability on large-scale trials to facilitate widespread commercial adoption. ECs and BCAs represent a significant step forward in postharvest technology, contributing to reduced food losses and enhanced global food security. Continued research and development, combined with strategic regulatory and consumer engagement, will be key to unlocking the full potential of this sustainable solution.

## Figures and Tables

**Table 1:** Matrices frequently used for edible coating dispersion and impact of fruit quality.

Fruit	Matrix	Additives	Coating Technique	Storage Condition	Finds/Benefits	References
Apple	Polyvinyl alcohol-native potato starch	Carvacrol	Dipping	25 °C, 65% RH, 14 days	Did not preserve fruit quality; exhibited high black and green mold control	[45]
Apple and Persimmon	Cassava starch-gellan gum	Thyme essential oil	Manual	25 °C, 65% RH, 14 days	Decreased water loss for persimmons and reduced incidence and severity of black spot and gray mold	[74]
Citrus	Hydroxypropyl-methylcellulose and beeswax	GRAS salts		5 °C, 90% RH, 28 and 56 days + shelf life	Reduce anthracnose severity and enhance CO_2_ without compromising sensory	[75]
Citrus	Carnauba wax nanoemulsion (CWN)	-	Manual	Cold storage + shelf life	Decreased water loss, increased CO_2_, and low O_2_ while producing less ethanol and volatile profile alterations	[8]
Citrus	Citrus pectin—beeswax	Essential oils	Dipping	20 °C, 80% RH, 14 days	Reduced weight loss and maintained firmness	[76]
Guava	Zein	Chitosan nanowhiskers	Dipping	26 °C, 65% RH, 8–11 days	Reduce *Colletotrichum* species severity while preserving quality and extending shelf life	[77]
Papaya	CWN–HPMC	Ginger oil	Manual	Shelf life, Cold storage + shelf life	Decreased water loss, color development, and ripening while showing positive effects against fungal diseases	[23]
Strawberry	Cassava starch	Sophorolipid fructooligosaccharides	Dipping	4 °C, 85% RH, 15 days	Delayed fruit senescence, mold, and yeast growth inhibition	[78]
Strawberry	Nanochitosan	Propolis	Dipping	4 °C, −RH, 8 days	Increased levels of total phenols, flavonoids, and antioxidant capacity	[79]
Strawberry, Papaya, Avocado, Banana	Egg-protein	Curcumin and cellulose nanocrystals	Dipping	−°C, −RH, 8–11 days	Reduced enzymatic browning, delayed pulp ripening, and maintained firmness	[66]
Tomato	CWN	-	Dipping	23 °C, 80% RH, 15 days	Decreased water loss, improved gloss, and higher consumer acceptance	[44]

(−) means not used or not cited.

**Table 2:** Research summary of using biocontrol agents into edible coatings dispersions for fruit preservation.

Antagonist	Fruit	Matrix and Additives	Target Microorganism	Storage Condition	Finds	Ref.
*Metschnikowia pulcherrima*	Apple	Apple residues	*Penicillium expansum*	21 °C, − % RH, 17 days	Reduced fungus growth and mycotoxin (patulin).	[116]
*Meyerozyma caribbica*	Avocado	Sodium alginate	*Colletotrichum gloeosporioides*	Cold storage + self-life, self-life	Decreased disease severity and incidence.	[40]
*Lacticaseibacillus casei* (Lbc1.5 or Lbc3)	Fresh pistachio fruit	Seed mucilage-based (*Lallemantia royleana* mucilage)	*-*	4 °C, 85% RH, 35 days	EC retarded mold and yeast growth, with a stronger effect when loaded with BCA. EC also preserved physicochemical and sensory properties.	[112]
*Lactobacillus rhamnosus* (L) or *Bifidobacterium animalis* subsp. *Lactis* (B)	Fresh-cut apple	Alginate + calcium chloride and prebiotics (inulin and oligofructose)	*Escherichia coli* *Listeria innocua*	5 °C, − % RH, 8 days	L + EC ^1^ and B + EC ^1^ reduced *E. coli* viability and showed bactericidal effects against *L. innocua.*	[107]
*Candida sake* (C)	Grape	Maltodextrin, skimmed milk and sucrose formulation (M), or pregelatinized potato starch (PS)	*Botrytis cinerea*	20 °C, 85% RH, 10 days	PS + C reduced fungal severity by over 65%, while M + C achieved a reduction of approximately 40%.	[114]
*C. sake*	Grape	HPMC ^2^, starch, sodium caseinate (SC) or pea protein	*B. cinerea*	20 °C, 85% RH, 7 and 12 days	EC enhanced *C. sake* survival and efficacy, while SC and starch were recommended for cost-effective control of *B. cinerea*.	[28]
*Lactobacillus plantarum* subsp. *plantarum* (LP)	Grape	Pregelatinized potato starch (PS) or sodium caseinate	*B. cinerea*	20 °C, 85% RH, 7 and 9 days	Coating minimally affected quality but boosted LP’s anti-fungal performance, while PS + LP showed the greatest incidence reduction	[37]
*Candida oleophila*	Grapefruit	Shellac-based free alcohol or morpholine; or sucrose ester and surfactants	*P. digitatum*	13 °C, − % RH, 30 and 90 days	Optimized EC improved yeast survival on fruit and boosted disease control.	[111]
*Wickerhamomyces anomalus* (W)	Oranges	Pectin + calcium chloride	*P. digitatum;* *P. italicum*	Inoculated fruits: 23 °C, 90% RH, 7 days. Quality: 5 °C, 90% RH, 60 days. Then: then: 20 °C, 75% RH, 7 days	Viability of W inserted into EC was maintained during storage and modified chilling injury index.	[109]
Bacteriophage cocktail (Phages: *S.* Enteritidis F5–4, *S.* Typhimurium L2–1 and *S.* Typhimurium ICB1–1)	Strawberries	Whey protein (WP), carboxymethyl cellulose, chitosan, or sodium alginate	*S. enterica* subsp. *enterica* serovars Enteritidis and Typhimurium	4 °C, 90% RH, 5 days	WP + phage preserved fruit quality and showed the highest antimicrobial activity against *S.* Enterica	[92]
*Bacillus subtilis* HFC103	Strawberries	Candelilla wax + guar gum	*Rhizopus stolonifer*	25 °C, − % RH, 6 days	Preserve fruit quality parameters, reduced moisture loss and fungal decay	[36]
Bacteriophage (V) (vB_EcoMH2W), *Caudovirales* order	Tomato	Chitosan-based commercialized by FreshSeal^®^ BASF Corporation, NJ, USA	*Escherichia coli*	20 °C, − % RH, 6 days	V + EC ^1^ reduced bacterial growth by three logarithmic units compared to chitosan coating alone.	[113]

^1^ refers to the edible coating (EC) used in the respective study, ^2^ hydroxypropylmethylcellulose (HPMC), and (−) means it is not cited.

## Data Availability

No new data were created or analyzed in this study. Data sharing is not applicable to this article.
